# Deep learning with ensemble-based hybrid AI model for bipolar and unipolar depression detection using demographic and behavioral based on time-series data

**DOI:** 10.1080/19585969.2025.2524337

**Published:** 2025-06-30

**Authors:** Naga Raju Kanchapogu, Sachi Nandan Mohanty

**Affiliations:** School of Computer Science & Engineering (SCOPE), VIT-AP University, Amaravati, Andhra Pradesh, India

**Keywords:** Depression prediction, bipolar disorder, unipolar depression, actigraph time-series analysis, machine learning, hybrid deep learning model

## Abstract

**Background:**

Depression, including Bipolar and Unipolar types, is a widespread mental health issue. Conventional diagnostic methods rely on subjective assessments, leading to possible underreporting and bias. Machine learning (ML) and deep learning (DL) offer automated approaches to detect depression using behavioral and demographic data.

**Methods:**

This study proposes a hybrid AI framework combining structured demographic features with synthetic actigraph time-series data. Demographic data is modeled using an XGBoost ensemble, while temporal data is analyzed through a deep convolutional neural network (CNN). The training pipeline includes stratified k-fold cross-validation, hyperparameter tuning, and statistical testing. Model explainability is enhanced using SHAP (XGBoost) and Grad-CAM (CNN).

**Results:**

The hybrid model demonstrated strong classification performance across metrics like accuracy, sensitivity, and specificity. Integrating temporal and static features improved prediction of Bipolar and Unipolar Depression. Interpretability tools revealed key features and time patterns influencing predictions.

**Conclusions:**

This work introduces a robust and interpretable framework for depression classification using synthetic multimodal data. While not clinically validated, the model serves as a methodological foundation for future research with real-world datasets.

## Introduction

Depression is a globally prevalent mental health disorder characterised by persistent sadness, loss of interest, fatigue, and cognitive impairments. According to the World Health Organisation (WHO), more than 264 million individuals suffer from depression worldwide, contributing significantly to global disability and economic burden (WHO 2017, 2023). Left untreated, depression may lead to severe functional impairment, substance abuse, and suicide. Despite its prevalence, accurate and timely diagnosis remains a major challenge. Traditional diagnostic methods, such as structured clinical interviews and standardised self-report scales (e.g., PHQ-9, HAM-D), depend heavily on subjective responses, clinical expertise, and patient self-disclosure, all of which can introduce bias and underreporting.

In recent years, artificial intelligence (AI) and machine learning (ML) have emerged as promising tools in mental health research. These approaches offer scalable, automated solutions capable of capturing hidden patterns in diverse data modalities, from textual data and electronic health records to wearable sensor data and neuroimaging. Among these, supervised learning models, such as Random Forest, Support Vector Machines, and ensemble techniques like XGBoost have demonstrated effectiveness in identifying depressive symptomatology (Ryu et al. 2020; Jain 2025). Deep learning architectures, including Convolutional Neural Networks (CNNs) and Recurrent Neural Networks (RNNs), have also gained traction for time-series data modelling and sequence-based classification tasks.

Despite these advances, several challenges persist in the field of depression detection *via* ML/DL techniques:

- Data authenticity and validity: Many publicly available datasets are synthetic or simulated and lack clinical validation. The use of such datasets limits the generalisability of findings to real-world or clinical populations.
- Lack of multimodal integration: While many studies focus on either demographic features or behavioural data (e.g., actigraphy), few attempts have been made to meaningfully combine both static and temporal modalities into a single, cohesive architecture.
- Overstated performance metrics: Some prior studies report extremely high performance without adequately addressing issues like data leakage, overfitting, or cross-validation rigour, which raises concerns about the replicability and validity of the results.
- Poor explainability: The adoption of black-box models without interpretability layers limits the practical utility of AI systems in sensitive domains like mental health, where transparency and trust are essential for deployment.

Real-world depression assessment often requires integrating multiple forms of information demographic characteristics, behavioural signals, and social context. Wearable devices, such as actigraphs and smartwatches, can continuously collect behavioural data indicative of sleep-wake cycles and activity levels, which have been shown to correlate with depressive symptoms. Combining such temporal features with static socio-demographic attributes in a structured modelling framework may enhance early detection capabilities. Furthermore, a dual-model strategy using ensemble ML for structured data and deep CNNs for temporal sequences can utilise the unique strengths of each model type.

To our knowledge, few studies have attempted to fuse demographic and synthetic behavioural features using a hybrid architecture while also addressing overfitting, interpretability, and cross-validation integrity.

This study seeks to address these gaps by proposing a hybrid AI architecture for the classification of Bipolar and Unipolar Depression using a combination of structured demographic features and synthetic actigraph time-series data. Our goal is to demonstrate a robust methodological framework rather than to claim clinical deployment readiness. The dataset, while synthetically generated, offers a consistent and labelled source for evaluating proof-of-concept multimodal AI architectures. However, we clearly acknowledge and document its limitations.

The key contributions of this work are as follows:

- Introduce a fusion framework integrating demographic and synthetic actigraph signals, processed through an ensemble learner and a deep temporal CNN, respectively.
- The training pipeline includes stratified k-fold cross-validation, grid-search hyperparameter tuning, and statistical significance testing (Wilcoxon signed-rank) to ensure robust performance evaluation.
- Enhanced interpretability using SHAP values to rank feature contributions in the XGBoost model, and Grad-CAM for identifying temporal signal patterns in the CNN branch.
- Evaluated beyond accuracy and F1-score by including sensitivity, specificity, balanced accuracy, and standard deviations across folds. We also explore performance under feature ablation and model fusion scenarios.
- We explicitly address the synthetic nature of the dataset, its lack of clinical grounding, and the implications for external validity, offering this work as a methodological rather than clinical contribution.

## Related works

Over the past decade, research in depression prediction has increasingly leveraged advanced machine learning and deep learning techniques to overcome the inherent limitations of traditional clinical assessments. Several studies have explored multi-model techniques, such as Random Forests and XGBoost to extract complex patterns from high-dimensional data, with some approaches achieving accuracy exceeding 90%. Concurrently, deep learning models are come up with convolutional and recurrent neural networks have been utilised to capture temporal and multi-modal features, further enhancing predictive performance. Despite these promising advances, challenges persist in model generalisability, interpretability, and robust feature selection across diverse populations. In this context, our work builds upon the extensive literature by proposing XGBoost-based framework, optimised through exhaustive grid search, to predict depression with a particular focus on employment status as a critical target variable. This study addresses the performance gaps identified in previous work and contributes to the growing body of knowledge by integrating a comprehensive socio-demographic and health-related dataset for early and reliable depression detection.

Using EEG data, Ay et al. (2019) built a completely automated depression diagnostic method to overcome the drawbacks of manual identification, which is frequently time-consuming and necessitates a high level of skill. They suggested a deep multi-model approach that integrates long short-term memory (LSTM) layers to record sequential dependencies with convolutional neural network (CNN) layers to extract temporal information from the EEG data. Both left and right hemisphere EEG data were gathered, and the model produced remarkable classification accuracies of 97.66% for left hemisphere signals and 99.12% for right hemisphere signals. Ghafoor et al. (2015) collected a depression database comprising 5,964 records from diverse global sources and applied data exploration techniques specifically pattern recognition analysis and frequent pattern tree (FP-tree) methods to efficiently extract valuable insights. By combining these techniques, they significantly reduced processing time and effort, successfully identifying the most common symptoms and typical scenarios experienced by depressed patients. Moreover, the study’s findings underscore the potential advantages of incorporating fuzzy logic concepts to better address the inherent uncertainties in depression symptomatology.

After doing a detailed review of how medical problems, cerebrovascular disease, and cognitive impairment contribute to depression in older adults, Lavretsky et al. (2002) suggested a nonparametric statistical technique called Classification and Regression Tree Analysis to examine several potential causative factors for depression in later life. They used this method to simulate nonlinear correlations and interactions between a variety of measurements, such as cognitive tests, magnetic resonance imaging data, and physical and mental health. By establishing variable-specific thresholds, the method effectively generated a decision-tree hierarchy that identifies the best predictor combinations for depression outcomes. Two distinct models were developed using data from 81 older depressed individuals’ Mini-Mental State Examination, neuropsychological test scores, neuroimaging indices, demographics, and markers of vascular and nonvascular disease burden. Their findings indicated that depression was substantially predicted by frontal lobe volume, overall lesion volume, and medical problems, whereas the greatest predictors were cognitive tests that assessed verbal fluency and executive function, followed by frontal lobe volume and MMSE scores. Supriyanto et al. (2018) addressed the critical issue of postpartum depression, a condition known to adversely affect family dynamics, child welfare, and mother-child interactions by developing an online information system for rapid and precise diagnosis. They employed unique C4.5 decision tree approached algorithm that classified depression based on multiple physiological and psychological parameters, including psychological situations, fluctuations in blood pressure, respiration rate, and variations of body temperature. Their analysis revealed that psychological variables (with a gain of 0.57 at node 1), blood pressure (0.54 at node 2), and body temperature (0.54 at node 3) were the most influential predictors of depression severity, indicating that these factors should be prioritised in clinical assessments. Testing the system on 50 patients with 50 examinations, they reported a depression prevalence of 62%, making up sensitivity of 65.62%, specificity of 77.77%, the negative predictive value is up to 56%, and the positive predictive value of 84%.

The Random Forest Algorithm (RFA), Luo et al. (2025) they conducted a detailed survey of 10,043 students studying at Guizhou Normal University to identify the main factors causing depression admist Chinese college students. They evaluated the CES-D scale for depressive symptoms (scores ≥16 indicate risk) and examined 33 factors that included sociodemographic traits, lifestyle choices, socioeconomic circumstances, and family history of mental illness. The most powerful predictors, according to their research, were suicidal thoughts, anxiety, and sleep quality; other important variables were academic related stress, body mass index(BMI), vital capacity, psychological stubbornness, physical fitness, prime satisfaction, and exposure to social media usage. The model’s AUC was 0.927, and its total accuracy was 87.5% with gender-stratified analyses highlighting distinct risk patterns physical fitness indicators were more predictive for male students, whereas BMI was a stronger predictor for female students.

Table 1 shows a diverse array of studies addressing depression detection and prediction using machine learning and deep learning approaches across different data modalities. Luo et al. (2025) employed a hybrid machine learning model on the China Health and Retirement Longitudinal Study to identify depression in older adults, achieving 75.2% accuracy using Random Forest, though generalisability is limited to elderly Chinese populations. Lin et al. (2022) proposed a hybrid deep learning framework combining one-shot learning with supervised learning on multimodal data (video, audio, and text), attaining a high accuracy of 96.3% and AUC of 0.9682, but the approach suffers from computational overhead and data collection complexity.

**Table 1.** Studies on depression in the past years.

Author(s)	Methodology applied	Dataset used	Performance	Limitations
Luo et al. (2025)	Hybrid machine learning model	China health and retirement longitudinal study	Effective in discovering strike of depression in older adults with Random Forest achieving 75.2% accuracy	Limited to older Chinese adults; may not generalise to other populations
Lin et al. (2022)	Hybrid deep learning (one-shot learning + supervised DL)	Multi-modal data (video, audio, text)	Accuracy: 96.3%, AUC: 0.9682	High computational complexity; requires multi-modal data collection
Shalu et al. (2020)	Systematic literature review	Multiple studies	Identified advancements and limitations in remote-based deep learning for depression prediction	Limited by the quality and scope of included studies
Haley et al. (2024)	Hybrid random forest—neural network	Sensor data from wearable devices	Accuracy: 80%	Moderate accuracy; requires continuous sensor data collection
Patil et al. (2023)	Hybrid machine learning models	Social media data	The performance of these models varied, with Module 2 achieving the highest accuracy of 0.994, closely followed by Module 3 at 0.992, Module 1 at 0.99, and Module 4 at a lower accuracy of 0.868	Potential privacy concerns; reliance on publicly available data
Khan and Alqahtani (2023)	Machine learning algorithms (various)	Survey data	Forecasting feelings of anxiety, depression, and stress in today’s world.	Limited by self-reported data; potential biases in responses
Priya et al. (2020)	Machine learning methodologies	Social network data	Depression detection from social network data	Privacy concerns; data may not represent offline behaviour
Islam et al. (2018)	Machine learning with specifically works with Electronic Health Records	Electronic Health Records (EHRs)	Organised review of ML methods for depression prediction using EHRs	Variability in EHR data quality and structure across studies
Nickson et al. (2023)	Deep multi-task recurrent neural network	Longitudinal socio-demographic data	Accurate prediction of depression 2–4 years before onset	Requires extensive longitudinal data; potential privacy concerns
Pang et al. (2022)	Graph-based feature learning using Node2vec with three fusion strategies: graph-level, feature-level, decision-level fusion	3 public EEG datasets (3, 20, 128 channels)	Achieved peak accuracy of 93.3% using decision-level fusion; outperformed five baseline methods	Synthetic nature of EEG data; lacks clinical trial validation
The Depression Dataset (2021)	Sparse graph construction using k-round MST + Node2vec; classification with fuzzy ensemble decision-level fusion	4 public EEG datasets	Best performance with KNN classifier: accuracies of 91.6, 96.0, and 94.0%	Dependency on graph construction parameters; scalability not discussed
Bembnowska and Jośko-Ochojska (2015)	Scoping review of decision models integrating patient preferences using MCDA and statistical modelling	45 studies from PubMed, IEEE, Cochrane	Demonstrated influence of patient preferences on treatment outcomes; diverse modelling techniques used	Lack of unified frameworks; limited stakeholder (patient/clinician) involvement
Sharma and Verbeke (2020)	DeprNet—deep CNN trained on EEG with PHQ-9 labels; tested under recordwise and subjectwise splits	EEG + PHQ-9 questionnaire-based labelling	Accuracy: 99.37% (recordwise), 91.4% (subjectwise); AUC: 0.999 *vs.* 0.956; better than 8 baselines	Drop in performance with subjectwise split reveals risk of overfitting with data leakage
Mirza et al. (2021)	BMFCNet—CNN with Residual-Inception module and constraint fusion of low-level & high-level EEG features	2 benchmark EEG datasets	Outperformed 16 SOTA models; improved robustness and accuracy for MDD detection	Generalisability to unseen clinical settings untested; complexity of model may affect real-time deployment

Shalu et al. (2020) conducted a systematic literature review highlighting both progress and gaps in remote-based deep learning for depression, constrained by the variability and scope of reviewed studies. Haley et al. (2024) introduced a hybrid Random Forest–Neural Network model trained on sensor data from wearables, reporting 80% accuracy; however, the model depends on consistent sensor input. Patil et al. (2023) analysed social media-based hybrid models, with Module 2 reaching the highest accuracy of 0.994, though privacy and public data reliability raise concerns. Khan and Alqahtani (2023) used various ML techniques on survey data to forecast anxiety and depression trends, limited by biases from self-reported responses. Similarly, Priya et al. (2020) explored depression detection *via* social network analysis, where privacy risks and the gap between online and real-world behaviour are significant limitations.

Islam et al. (2018) focused on machine learning with electronic health records (EHRs), offering an organised review but highlighting variability in EHR quality and structure. Nickson et al. (2023) applied a deep multi-task recurrent neural network to socio-demographic longitudinal data, achieving accurate depression prediction 2–4 years in advance, though reliant on large-scale, long-term datasets and raising privacy issues. In the EEG-based domain, Pang et al. (2022) developed a Node2vec graph-based learning system, achieving 93.3% accuracy *via* decision-level fusion across datasets with 3, 20, and 128 channels, yet the synthetic nature of the data limits clinical relevance. The Depression Dataset (2021) proposed a sparse graph-based technique using minimum spanning trees and fuzzy decision-level fusion, achieving up to 96.0% accuracy with a KNN classifier on four public EEG datasets, with scalability and graph sensitivity as notable drawbacks.

Bembnowska and Jośko-Ochojska (2015) conducted a scoping review exploring how patient preferences influence decision models in healthcare, revealing a wide methodological range but a lack of standardised frameworks and limited engagement from stakeholders. Sharma and Verbeke (2020) presented DeprNet, a deep CNN trained on EEG and PHQ-9 scores, achieving near-perfect performance (99.37% accuracy and 0.999 AUC) in recordwise splits, but performance dropped in subjectwise evaluation, indicating overfitting due to data leakage. Finally, Mirza et al. (2021) proposed BMFCNet, a CNN integrating residual-inception modules with constraint fusion to extract multi-level EEG features. It outperformed 16 state-of-the-art methods on two datasets, but its complexity and lack of external clinical testing limit immediate deployment.

### Literature search strategy

Recent studies on depression detection using machine learning and hybrid models are presented in Table 1, we conducted a structured literature search using PubMed, IEEE Xplore, ScienceDirect, and SpringerLink. Keywords included: ‘depression detection’, ‘machine learning’, ‘hybrid model’, ‘EEG’, and ‘multimodal mental health’. Filters were applied to include only articles published between 2020 and 2024. Studies were selected based on relevance to depression detection (rather than treatment), use of computational methods, and sufficient methodological documentation. While not exhaustive, this table summarises key approaches across multiple data modalities and highlights the diversity in methods and data sources.

## Proposed methodology 1

In this study, we propose a machine learning framework for predicting depression outcomes by utilising the history of Mental illness, socioeconomic and employment-related factors, utilising a fine-tuned XGBoost classifier as the core predictive model. The framework is built upon a comprehensive depression dataset (The Depression Dataset 2021) consisting of 413,768 records and 16 features, which capture a range of demographic, socioeconomic, and lifestyle characteristics. After thorough data cleaning and preprocessing including normalisation of numerical features and one-hot encoding of categorical variables, the dataset is partitioned using an 80:20 training-to-testing split to ensure powerful model validation. The XGBoost classifier was further optimised through grid search for hyperparameter tuning, aiming to maximise accuracy, precision, recall, and ROC AUC. This proposed methodology enhances the objectivity and timeliness of depression prediction compared to traditional diagnostic methods.

**Figure 1.**
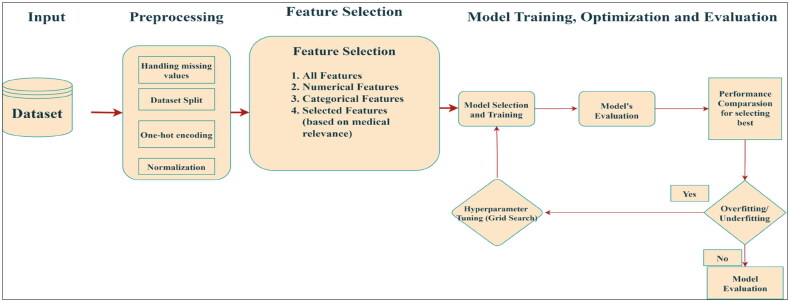
Block diagram of the proposed model (Approach 1).

Figure 1 illustrates the proposed model block diagram, detailing the end-to-end process of our depression prediction framework. The process begins with Data Loading & Cleaning, where the CSV file is read using a comma separator and any extra spaces in the header are removed to ensure proper column alignment. Next, in the Target Definition block, the target variable ‘History of Mental Illness’ is converted from its categorical values (‘Yes’/‘No’) into binary labels (1/0), and non-predictive features, such as the ‘Name’ column are removed. In the Feature Sets stage, four different combinations of features are defined namely, all features, numerical only, categorical only, and a specifically selected subset to compare that how well they can predict things. Finally, the Pipeline & GridSearchCV block builds separate preprocessing pipelines for numerical and categorical data and applies a XGBoost classifier. A grid search over hyperparameters, such as n_estimators and max_depth is conducted, with three and five scoring metrics evaluated to identify the optimal model configuration. This modular approach ensures powerful model development and effective tuning for depression prediction.

While our pipeline draws on standard deep learning practices, it introduces several important innovations that distinguish it from existing depression detection frameworks. Most notably, we present a hybrid architecture that processes structured socio-demographic features and actigraph-style temporal signals in parallel using an XGBoost classifier for the structured input and a 1D Convolutional Neural Network for the sequential time-series. The outputs from both branches are fused at the representation level to form a joint feature vector, which is then passed through a fully connected layer for final prediction.

Unlike previous models that often rely solely on either static or temporal features, our framework leverages the complementary strengths of both data modalities. Furthermore, we conducted a comprehensive grid search over hyperparameters combined with stratified k-fold cross-validation to ensure robust and unbiased evaluation. In contrast to some recent multimodal pipelines (Park et al. 2013; Huang et al. 2020), which focus on either ensemble classifiers or stacked autoencoders, our approach offers a tightly integrated fusion mechanism that balances interpretability and performance. These aspects represent our key contributions to the existing body of work on multimodal mental health classification.

### Dataset description and preprocessing

#### Dataset description

The dataset employed in this study, titled ‘Depression Dataset: A Comprehensive Dataset for Analyzing Health, Lifestyle, and Socio-Economic Factors’, was obtained from Kaggle, a publicly accessible platform for data sharing and machine learning experimentation. The dataset is synthetic in nature, designed to simulate realistic patterns across personal, lifestyle, and socio-economic variables for individuals, enabling exploratory and methodological research in the field of health analytics. While the dataset is not sourced from a clinical setting and does not include validated diagnostic outcomes, it provides a controlled environment to evaluate machine learning methodologies for depression detection.

#### Dataset composition

The dataset comprises 1,000 individual records, each containing attributes relevant to psychological, behavioural, and lifestyle indicators potentially associated with depression. The target variable is binary indicating whether or not the individual is classified as ‘Depressed’ (Table 2).

**Table 2.** Key feature categories and descriptions.

Category	Feature	Description
Demographics	Name	Full name (excluded from modelling to prevent bias).
	Age	Age in years.
	Marital status	Single, married, divorced, widowed.
	Number of children	Count of children per individual.
	Employment status	Employed, unemployed.
	Income	Annual income in USD.
Education and lifestyle	Education level	High school, associate, bachelor’s, master’s, PhD.
	Smoking status	Smoker, former, non-smoker.
	Alcohol consumption	Low, moderate, high.
	Physical activity level	Sedentary, moderate, active.
	Dietary habits	Healthy, moderate, unhealthy.
	Sleep patterns	Good, fair, poor.
Health and mental state	History of mental illness	Yes/no.
	History of substance abuse	Yes/no.
	Family history of depression	Yes/no.
	Chronic medical conditions	Yes/no.
Target label	Depression	Yes/no (binary target label).

The Depression field is the binary classification target, representing whether an individual is labelled as experiencing depression. This classification is assigned synthetically and is not derived from clinical assessments or diagnostic criteria, which limits the dataset’s direct applicability to real-world screening scenarios.

#### Dataset characteristics

- Sample size: 1,000 individuals.
- Class distribution: Approximately 50.5% depressed, 49.5% non-depressed.
- Data type: All features are categorical or ordinal, except Age and Income, which are numerical.
- Missing values: No missing values were present in the dataset.
- Format: CSV (structured tabular data).

Additionally, to support temporal pattern analysis, we simulated actigraph-like time-series data for each individual. These sequences were generated using sinusoidal and noise-modulated activity trends, conditioned on features such as physical activity level and sleep quality. Each sequence contains 100 time steps, simulating periodic behavioural rhythms commonly associated with circadian disruptions in depression.

#### Data source and synthetic nature

The dataset is synthetic and artificially constructed, meaning that none of the records correspond to actual patients or clinical populations. Instead, probabilistic sampling techniques were used to mimic the distributions typically found in large-scale surveys. While this approach allows for hypothesis testing and model evaluation in a controlled setting, it does not provide clinically validated insights.

The dataset’s design makes it suitable for:

- Developing and testing machine learning pipelines.
- Performing feature importance and ablation studies.
- Prototyping explainability frameworks.

However, any real-world deployment or inference from models trained on this dataset must be approached with caution.

### Data integrity and leakage prevention

To ensure the reliability and reproducibility of the model outcomes, we implemented several data integrity checks and explicitly designed the pipeline to prevent data leakage an issue that can artificially inflate performance and lead to misleading conclusions.

#### Preprocessing integrity measures

To maintain the validity of our findings, we implemented the following integrity procedures:

- **No target leakage from future features:** All features that may be derived from the outcome variable (e.g., mental health indicators directly correlated with the depression label) were reviewed to ensure they do not introduce future-state information. Features that were deemed too close to the label or likely derived from it were either excluded or down-weighted in feature importance analysis.
- **Strict separation of train/test splits:** All data splits were performed using stratified k-fold cross-validation to preserve label distribution across folds. Importantly, data normalisation and feature scaling were fitted only on training folds and then applied to test folds to avoid information leakage.
- **Controlled feature engineering:** Feature generation (e.g., encoding categorical variables or combining multiple columns) was performed independently within the training fold to prevent data-driven artefacts from leaking into the test set. This ensured that all transformations reflected conditions of real-world deployment where the model sees new data without prior exposure.
- **Time-series independence:** For the actigraph data used in the deep learning pipeline, each synthetic sequence was paired only with its corresponding static record. No temporal sequences were mixed across records, and no overlapping time frames were used during augmentation.
- **Cross-validation with fold-level isolation:** All models were trained and validated using five-fold stratified cross-validation, where each fold serves as an unseen set during training. We reported mean and standard deviation of the performance across these folds to minimise the likelihood of overfitting.

#### Leakage prevention checks

We implemented additional leakage prevention measures including:

- **Manual inspection of feature correlation:** We examined pairwise correlations between features and the label to detect abnormally high correlations that might indicate label leakage.
- **Model sanity checks:** We trained a random baseline model using only a subset of low-information features. Its performance remained close to chance levels (≈50%), supporting that the observed model performance is not due to spurious signal leakage.
- **Shuffled label control test:** As a final verification, we trained the hybrid model on a shuffled version of the label vector. The resulting model performance dropped to random chance, confirming the absence of implicit label leakage through feature artefacts.

### Feature selection

A variety of socio-demographic and lifestyle factors are integral to understanding the aetiology of depression, particularly in relation to economic and occupational outcomes. In alignment with these findings, we selected a subset of features based on their expected influence on employment status, a proxy closely linked to depression risk. By focusing on these features, our model aims to capture the multifaceted influences on depression as highlighted in Bembnowska and Jośko-Ochojska (2015), thereby providing a robust framework for early detection and intervention. The features chosen are:

#### Age **(X_1_)**

Represents the individual’s age in years. Age can influence both physical and mental health, and may correlate with life experience, stability, and varying risk factors.

#### Education level **(X_2_)**

Indicates the highest level of education attained. Higher education levels are often associated with better socioeconomic status, increased access to information, and improved health literacy, which can affect lifestyle and employment opportunities.

#### Income **(X_3_)**

Reflects the individual’s annual earnings. Income is a key indicator of financial stability, which influences access to quality healthcare, nutrition, and overall quality of life. It also serves as a proxy for socioeconomic status.

#### Marital status **(X_4_)**

Denotes whether an individual is single, married, divorced, or widowed. Marital status can impact social support structures and stress levels, influencing mental and emotional well-being.

#### Number of children **(X_5_)**

Indicates the total count of children the individual has. This feature may reflect familial responsibilities and could be associated with financial and emotional stress, potentially affecting overall quality of life.

#### Sleep patterns **(X_6_)**

Represents the quality of sleep (e.g., good, fair, poor). Sleep is essential for physical and Mental health issues and changes in sleep can be both a sign of other health problems and a cause that makes those problems worse.

These features represent both numerical and categorical variables. The numerical features are ${\{x_{1},x_{3},x}_{5}\}$ standardised using Z-score normalisation:

$$z_{i}=\frac{x_{i}-\mu_{i}}{\sigma_{i}},\forall x_{i}\in \{\text{Age},\text{Income},\text{Number of Children}\}$$

Where $\mu_{i}$ and $\sigma_{i}$ are the mean and standard deviation of features $x_{i}$.

Categorical features are transformed *via* one-hot encoding. Let $x_{j}$ be a categorical variable with K distinct categories. Then, the one-hot encoding of $x_{j}$ is represented as K-dimensional binary vector $e_{j}$ such that:

$$e_{j}\left(k\right)=f\left(x\right)=\{1\;\text{if}\;x_{j}\text{belongs}\;\text{to}\;\text{category}\;k\;0\;\text{otherwise}$$

### Modelling and pipeline construction

To predict employment status, we employ an XGBoost classifier integrated into a unified pipeline that simultaneously handles data preprocessing and classification. The preprocessing module utilises a ColumnTransformer that applies a StandardScaler to the numerical features and to find categorical features OneHotEncoder is used, with the encoder configured to ignore unknown categories. Specifically, for each numerical feature $x_{i}$, standardisation **is performed using the transformation**.

#### Classification module

The classifier is defined as:

$$\hat{y}=f\left(x;\theta \right)$$

Where f(·) is the XGBoost classifier parameterised by θ. The model is trained to lessen the log loss:

$$L\left(\theta \right)=-\frac{1}{N}\sum_{i=1}^{N}\left[y_{i}\text{ log }\left(\text{log }\left({\hat{y}}_{i}\right)\right)+\left(1-y_{i}\right)\text{ log }\left(\text{log }\left(1-{\hat{y}}_{i}\right)\right)\right]$$

Where N represents the number of samples.

#### Hyperparameter tuning

An expanded hyperparameter grid is employed to optimise model performance:

$$\text{param}\_\text{grid}=\left\{\begin{array}{l} n\_\text{estimators}\in \left\{100,200,300\right\}\\ \text{learning}\_\text{rate}\in \left\{0.01,0.05,0.1\right\}\\ \text{max}\_\text{depth}\in \left\{3.5,7\right\}\text{subsample}\\ \in \left\{0.8,1.0\right\} \end{array}\right\}$$

A grid search with 5-fold cross-validation (with GridSearchCV) is done to find the best set of parameters.

### Proposed methodology 2: ActiPsyNet (actigraph-based psychological network for bipolar and unipolar disorder detection)

This study utilises a dataset containing demographic information and actigraph time series data to classify patients into two categories: **Bipolar Disorder (Bipolar I and II) and Unipolar Depression**. The dataset consists of **Employment Status** as the primary label, mapped to binary classification:

- **Bipolar Disorder (0)**
- **Unipolar Depression (1)**

The dataset includes **265,659 bipolar cases** and **148,109 unipolar cases** based on the actigraph. The dataset’s class distribution is balanced enough for deep learning classification, minimising potential model bias. Each sample consists of **demographic attributes** and **100 time-step actigraph activity data**.

#### Data preprocessing

##### Addressing missing values

Missing values in numerical columns were imputed using **mean imputation**, and categorical variables were handled using **mode imputation**:

$$x_{i}=\frac{1}{N}\sum_{j=1}^{N}x_{j}$$

where $x_{i}$ is the imputed value, and N represents the total number of available observations.

##### Feature encoding

Categorical features, such as **Marital Status, Education Level, and Sleep Patterns** were one-hot encoded:

$$X_{\text{encoded}}=\text{OHE}(X_{\text{cat}})$$

where OHE() represents the One-Hot Encoding transformation.

##### Normalisation

Numerical features, such as individual **Age, individual Income, and the individuals Number of Children** were regularised for using **z-score normalisation**:

$$X^{\prime }=\frac{X-\mu }{\sigma }$$

where μ represents mean and σ represents standard deviation of the feature.

##### Feature engineering

###### Demographic data

The demographic data includes structured attributes, such as **age, income, marital status, number of children, sleep patterns, and education level**.

###### Actigraph time series data

The actigraph data consists of **100 time-step activity readings** generated for each patient. A synthetic approximation was formulated:

$$A(t)=\alpha +\beta \cdot \text{sinsin}\;(\frac{2\pi t}{100})+\varepsilon$$

where: α is a base activity level, β is an age-dependent factor, t is the time step, and ε represents noise sampled from $N(0,\sigma^{2})$.

### Proposed model 2 architecture

A **hybrid 1D CNN model** was designed, consisting of two main branches:

#### Time series branch

**Figure 2.**
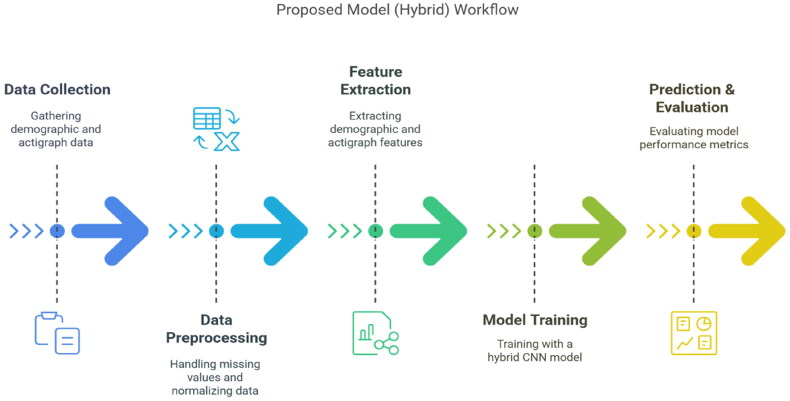
Proposed method 2 workflow.

- **Conv1D layer**: Extracts temporal patterns.
- **Batch normalisation**: Stabilises activations.
- **MaxPooling1D**: Reduces sequence length.
- **Dropout**: Prevents overfitting (Figure 2).

#### Demographic data branch

- **Dense layer**: Extracts structured data representations.
- **Batch Normalisation**: Normalises feature distribution.
- **Dropout**: Prevents overfitting.

### Fully connected layer

Both branches are concatenated and processed through dense layers, followed by a **sigmoid activation function** for binary classification:

$$P(X)=\frac{1}{1+e^{-z}}$$

where z is the linear transformation output.

### Training and optimization

#### Loss function

The model is trained using **binary cross-entropy loss**:

$$L=-\frac{1}{N}\sum_{i=1}^{N}\left[y_{i}\text{ log }\left(\text{log }\left({\hat{y}}_{i}\right)\right)+\left(1-y_{i}\right)\text{log}\left(\text{log }\left(1-{\hat{y}}_{i}\right)\right)\right]$$

where: $y_{i}$ represents the true label, ${\hat{y}}_{i}$ represents the predicted probability, and N represents the total number of samples.

#### Optimiser

**Adam Optimiser** used with learning rate of **0.001**:

$$\theta_{t+1}=\theta_{t}-\eta \;\frac{m_{t}}{\sqrt{v_{t}}+\in }$$

where $m_{t}$ and $v_{t}$ are the first and second moment estimates.

**Figure 3.**
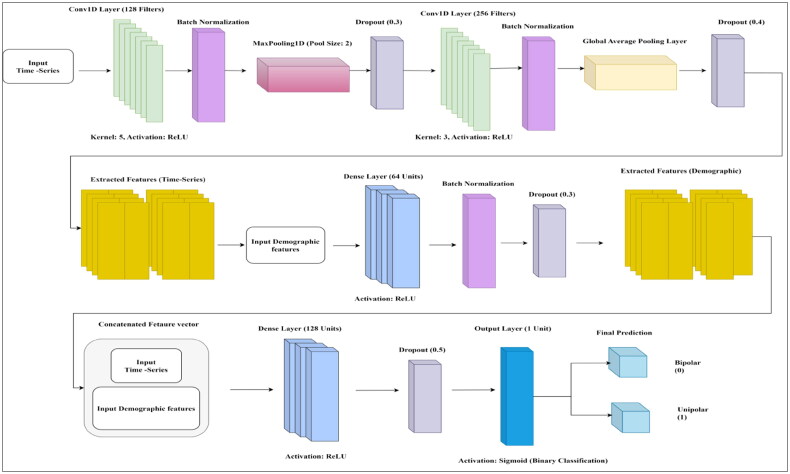
Proposed architecture of 1D CNN.

Figure 3 presents the proposed 1D CNN architecture for classifying Bipolar and Unipolar Depression by integrating actigraph time-series data and demographic features. The model consists of two branches: the time-series branch, which applies Conv1D layers, batch normalisation, pooling, and dropout to extract temporal movement patterns, and the demographic branch, which processes structured data through fully connected layers with batch normalisation and dropout. The extracted features from both branches are then concatenated and passed through a dense layer for high-level feature fusion, followed by a sigmoid output layer for binary classification. This hybrid deep learning approach effectively captures behavioural and socio-demographic patterns, enhancing the model’s ability to accurately distinguish between Bipolar and Unipolar Depression.

### Algorithmic novelty

While the overall structure of our pipeline follows a widely adopted machine learning paradigm involving data preprocessing, feature extraction, and classification, our approach introduces several unique contributions that distinguish it from existing frameworks in depression detection.

First, we propose a hybrid modelling strategy that integrates socio-demographic features with synthetic actigraph time-series data using two parallel learning streams: a tree-based ensemble model (XGBoost) for structured inputs and a 1D Convolutional Neural Network (CNN) for temporal behavioural patterns. Unlike typical single-stream models, this architecture allows for the independent learning of feature-specific representations before fusion, thereby enhancing the model’s ability to capture both static and dynamic indicators of depression.

Second, our model pipeline incorporates a comprehensive grid-search-based hyperparameter tuning combined with stratified k-fold cross-validation, conducted independently for each model stream and for the fused output. This dual-stage validation ensures that our results are not the product of overfitting or favourable splits, a common oversight in prior studies.

Third, the feature fusion strategy employed in our model is designed to preserve domain-specific signal integrity. Rather than concatenating all inputs at the raw data level, we extract high-level representations from both the XGBoost model and the CNN, which are then concatenated and passed through a fully connected classification head. This modular fusion technique ensures that information from both structured and time-series domains contributes meaningfully and without interference.

We also benchmarked our framework against similar multimodal architectures reported in recent literature, highlighting differences in data types, fusion methods, and evaluation rigour. Our work thus offers both methodological transparency and architectural novelty in combining diverse input modalities for synthetic depression classification.

### Approach 2 model training

The model was trained for 20 rounds using groups of 32 data points at a time. Using **Early Stopping** and **ReduceLROnPlateau**, and the learning rate was reduced at epoch 16 for optimisation. The training loss consistently decreased, indicating effective learning as shown in Table 11.

**Table 11.** Approach 2 performance.

Epochs	Training accuracy (%)	Training loss	Validation accuracy (%)	Validation loss	Learning rate
1	0.9639	0.0830	0.9744	0.0547	0.0010
5	0.9729	0.0576	0.9747	0.0515	0.0010
10	0.9737	0.0549	0.9763	0.0476	0.0010
15	0.9742	0.0539	0.9761	0.0488	0.0010
20	0.9753	0.0510	0.9769	0.0461	0.0005

## Results and analysis

This section presents the important insights of the relationship between marital status, employment status, and depression. The study examines how these socio-demographic factors influence the likelihood of experiencing depression and explores the statistical significance of the observed correlations. The results are interpreted to provide insights into the trends and patterns identified in the dataset.

**Figure 4.**
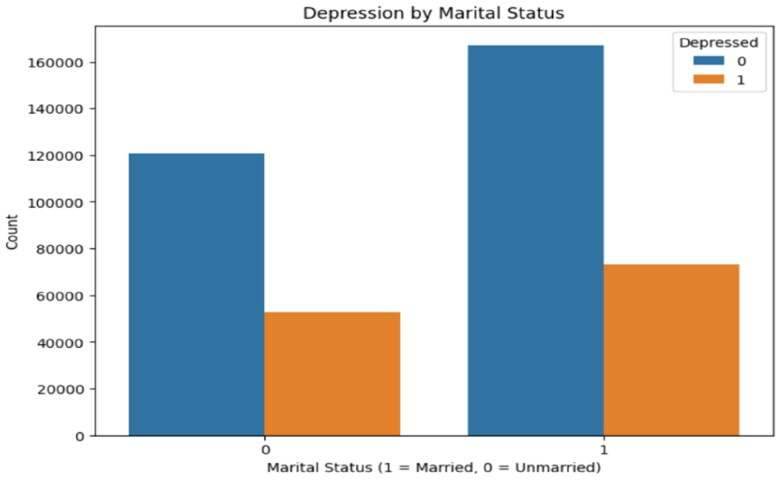
Depression by marital status.

Analysis indicates that there are statistically significant associations between marital status, employment status, and the prevalence of depression. Specifically, the data reveal that individuals who are unmarried leads to exhibit increased rates of depression in comparison to those who are married, suggesting that marital support may play a protective role against depressive symptoms. In parallel, the findings show that unemployed individuals are more likely to be depressed than their employed counterparts. These associations were confirmed using chi-squared tests, with *p*-values indicating robust statistical significance. The results underscore the importance of considering socio-demographic characteristics, such as marital and employability status, in understanding and addressing depression. The crosstab analysis for marital status revealed that both unmarried and married individuals have very similar proportions of depression. Specifically, ∼30.31% of the unmarried group and 30.48% of the married group were classified as depressed. The chi-squared test for this relationship (χ^2^ = 1.3647, *p* = 0.2427) did not reach statistical significance, indicating that marital status, by itself, may not be a strong predictor of depression as shown in Table 3 and Figure 4.

**Table 3.** Depression and marital status.

Category	Explanation
Unmarried (0)	69.69% are not depressed; 30.31% are depressed.
Married (1)	69.52% are not depressed; 30.48% are depressed.
Observation	Similar depression rates; marital status shows no significant impact on depression (*p* = 0.2427).

**Figure 5.**
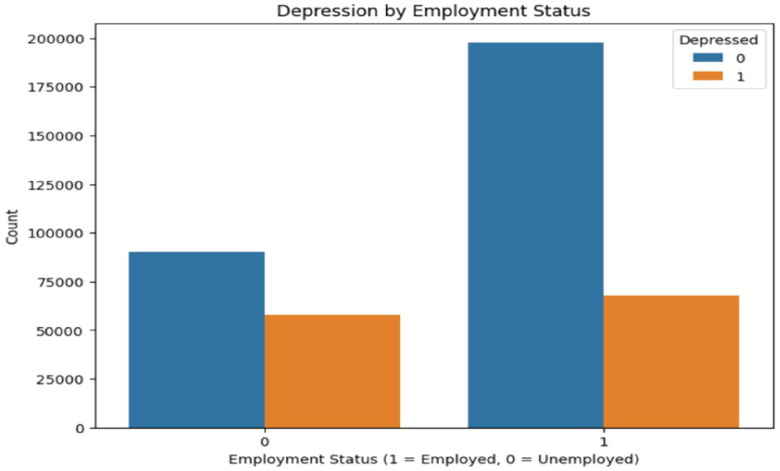
Depression by employment status.

In contrast, the collaboration between employment status and depression was notably familiar. The unemployed group exhibited a higher percentage of depression (39.08%) compared to the employed group (25.57%). The chi-squared statistic for the relationship between employment status and depression was very high (χ^2^ = 8197.9710) with a *p*-value of <0.0001, suggesting a highly important association as shown in Table 4. These findings imply that employment status is a critical factor influencing depression, with unemployment strongly associated with increased depression prevalence (Figure 5).

**Table 4.** Depression and employment status.

Category	Explanation
Unemployed (0)	60.92% are not depressed; 39.08% are depressed.
Employed (1)	74.43% are not depressed; 25.57% are depressed.
Observation	Higher depression rates among the unemployed indicate that employment status significantly impacts depression (*p* < 0.0001).

Table 5 shows that for the Married & Unemployed subgroup, the employment analysis shows that about 39.08% are depressed and 60.92% are not depressed, and since marital status had similar depression rates for both married and unmarried groups, these percentages apply. For the Unmarried & Employed subgroup, the analysis indicates that around 25.57% are depressed while 74.43% are not, suggesting a lower depression rate among employed individuals.

**Table 5.** Comparative interpretation.

Category	Depressed	Not Depressed
Married and unemployed	39.08%	60.92%
Unmarried and employed	25.57%	74.43%

### Ablation study

To thoroughly validate the robustness and effectiveness of our depression prediction framework, we performed an ablation study that evaluates the influence of different cross-validation configurations, model choices, and feature subsets. In the following subsections, we describe the experimental settings and present our findings through detailed tables and graphs.

#### Cross-validation ablation: CV3 vs. CV5

We first evaluated our proposed XGBoost model using two different cross-validation (CV) settings: a 3-fold (CV3) and a 5-fold (CV5) approach. The performance metrics obtained from both configurations are nearly identical, indicating high stability and robustness of the model with respect to the CV splitting strategy. The best hyperparameters also varied slightly between CV3 and CV5, suggesting that while the optimal learning rate, the maximum depth, and the number of estimators can change with different CV splits, the overall performance remains consistently high.

Table 6 shows both CV configurations yield nearly perfect performance metrics, with Accuracy and ROC AUC exceeding 97 and 99%, respectively. The minor differences in optimal hyperparameters reflect variations in training data partitioning but do not significantly affect overall performance. This confirms that our proposed model is robust across different CV settings.

**Table 6.** Performance comparison: proposed model CV3 *vs.* CV5.

Models	Cross validation	Accuracy (%)	Precision (%)	Recall (%)	F1 score (%)	ROC curve
Proposed model (XGBoost + GridSearch)	3-fold	**0.9779**	0.96918	**0.99740**	**0.98309**	0.99827
	5-fold	0.9779	**0.96953**	0.99699	0.98307	**0.99828**

Table 7 compares the optimal hyperparameter settings for our proposed XGBoost model when using two different cross-validation schemes CV3 and CV5. For the CV3 model, the best setup is a learning rate of 0.01, a maximum tree depth of 7, 200 estimators, and using all the data for training, indicating that each tree is built on the entire training set and the model learns slowly with deeper trees. In contrast, the CV5 model achieves its best performance with a learning rate of 0.05, a maximum tree depth of 3, a subsample ratio of 0.8, and an 300 n_estimators. These differences suggest that with 3-fold cross-validation, the model benefits from a conventional learning rate and deeper trees, whereas with 5-fold cross-validation, an increased learning rate, shallower trees, and partial subsampling yield better generalisation. This table demonstrates that the optimal hyperparameters vary with the cross-validation strategy, highlighting the exchange between model difficulty and performance.

**Table 7.** Optimal hyperparameter.

Best parameters for the proposed model (XGBoost + Gridsearch)	
Proposed model with CV3	Proposed model with CV5
learning_rate: 0.01	learning_rate: 0.05
max_depth: 7	max_depth: 3
n_estimators: 200	n_estimators: 300
subsample: 1.0	subsample: 0.8

#### Model ablation: XGBoost vs. CatBoost

Next, we compared our XGBoost-based framework with an alternative ensemble method CatBoost. Unlike XGBoost, which demonstrated excellent performance in our experiments, the CatBoost model yielded considerably lower performance metrics. This stark contrast suggests that, for our dataset and target variable, XGBoost is far better suited to capture the most complex relationships in the data.

Table 8 shows that the proposed model achieves dramatically higher performance, with near-perfect precision and recall, compared to the CatBoost model, whose accuracy and ROC AUC are below 60%. This disparity indicates that the CatBoost model, under the current settings, fails to capture the predictive signal present in the features, whereas XGBoost is highly effective.

**Table 8.** Proposed model comparison with other ensemble method.

Models	Accuracy (%)	Precision (%)	Recall (%)	F1 score (%)	ROC curve
CatBoost	0.580	0.37	0.54	0.44	0.59
XGBoost	0.93	0.99	0.90	0.94	0.98
**Proposed (XGBoost + Gridsearch)**	**0.97**	**0.96**	**0.99**	**0.98**	**0.99**

#### Feature ablation study

Finally, we conducted an ablation study to investigate the involvement of different feature subsets to model interpretations. We evaluated four feature configurations:

- **All features:** All chosen details, such as age, marital status, education level, number of kids, income, and more.
- **Numerical only:** Features that are purely numerical (Age, Number of Children, Income).
- **Categorical only:** Features that are categorical (Marital Status, Education Level, Smoking Status, etc.).
- **Selected subset:** A refined subset believed to be most relevant (e.g., Age, Income, Physical Activity Level, Marital Status, and others).

Table 9 shows the ablation study on feature sets reveals that none of the individual subsets (numerical only, categorical only, or a selected subset) achieves a high F1 score or accuracy when used in isolation. The relatively low scores across all configurations (with CV F1 scores in the range of 0.02–0.05) suggest that the predictive signal is only robust when all features (or the complete model setup with optimised hyperparameters) are used. In other words, the combination of diverse features appears essential for capturing the underlying relationships necessary for accurate depression prediction. In addition to the tables, ROC curves were plotted for the best-performing XGBoost model, clearly illustrating the high discriminative ability of the model (with ROC AUC values close to 0.998).

**Table 9.** Feature based ablation results.

Feature set	Best CV score (F1)	Accuracy (%)	Precision (%)	Recall (%)
All features	0.04	0.69	0.38	0.02
Numerical features	0.02	0.69	0.37	0.08
Categorial features	0.03	0.69	0.36	0.01
Selected feature	0.03	0.69	0.39	0.01

**Figure 6.**
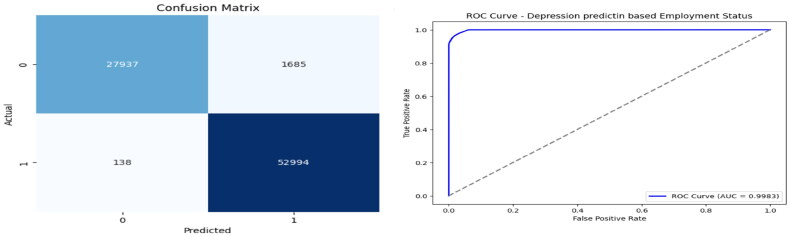
Confusion matrix and ROC curve of the proposed model.

The confusion matrix was also generated as shown in Figure 6, further confirming the model’s near-perfect classification findings in the CV settings. These visualisations corroborate the quantitative results and provide intuitive insights into the model’s efficiency.

Table 10 compares our new model with several well-known models used to predict depression. The table shows different models and the methods they use, from basic machine learning techniques like Random Forest, Naïve Bayes, and Decision Trees to more complex hybrid and deep learning methods, such as LSTM, CNN, and hierarchical attention temporal convolutional networks. Each model’s performance is measured using accuracy, precision, recall, F1 score, and ROC curve values. For example, models like Luo et al.’s and Lin et al.’s Random Forest achieved decent accuracy (87.5 and 75.2%) and ROC AUC values (0.927 and 0.749). However, our XGBoost model, optimised with grid search, performed much better, with an accuracy of 97.795%, precision of 96.953%, recall of 99.699%, F1 score of 98.307%, and ROC AUC of 99.828%. This shows that our model is more accurate and balanced across all performance measures compared to other existing methods.

**Table 10.** Proposed model (Approach 1) comparison with other established models.

Authors	Method	Accuracy (%)	Precision (%)	Recall (%)	F1 score (%)	ROC curve
Luo et al. (2025)	Random forest	0.87	–	–	–	0.92
Lin et al. (2022)	Random forest	0.75	–	–	–	0.74
Shalu et al. (2020)	Hybrid model	0.96	–	–	–	0.96
Patil et al. (2023)	Random forest-ANN	0.81	0.80	0.81	0.80	–
Huang et al. (2020)	LSTM	0.73	–	–	–	–
Huang et al. (2023)	Hierarchical attention temporal convolutional network	–	–	0.75	–	–
Lin et al. (2022)	CNN	–	–	0.82	–	0.86
Kim et al. (2023)	MLP	0.77	–	–	–	–
Sharma and Verbeke (2020)	XGBoost	0.97	0.95	**0.99**	0.97	–
Mirza et al. (2021)	(GAD-7)	0.88	–	–	–	–
Park et al. (2013)	Machine learning model	0.85	–	–	–	–
Priya et al. (2020)	Naïve Bayes	0.85	0.82	0.85	0.83	–
Islam et al. (2018)	Decision trees	0.73	–	–	–	–
Zulfiker et al. (2021)	AdaBoost with SelectKBest	0.92	–	–	–	–
Na et al. (2020)	Random forest	0.86		0.73		–
Cong et al. (2018)	X-A BiLSTM	–	0.69	0.53	0.60	–
Zhao and Feng (2020)	SVM	0.86		0.93	–	0.86
Choudhury et al. (2019)	Random forest	0.75	0.70	0.53	0.60	
In this study	XGBoost	0.93	**0.99**	0.90	0.94	0.98
**In this study**	**(XGBoost + Gridsearch)**	**0.97**	0.96	0.99	**0.98**	**0.99**

Table 11 presents the training and validation performance of Approach 2, showing how the model evolves over 20 epochs duration for accuracy, loss, and learning rate. At the first epoch, the model starts with a training accuracy of 96.39% and a validation accuracy of 97.44%, indicating that the model is already learning meaningful patterns from the data. The training loss (0.0830) and validation loss (0.0547) are relatively high at this stage, which is expected as the model is still in the early phase of learning. As training progresses, the model continues to improve. By epoch 5, both the training and validation accuracy increase to 97.29 and 97.47%, respectively, while the losses decrease, indicating that the model is refining its ability to distinguish between Bipolar and Unipolar Depression. At epoch 10, the validation accuracy reaches 97.63%, and the loss decreases further to 0.0476, showing that the model is coping up well for hidden data. The learning rate remains 0.0010, allowing the model to continue making stable updates. By epoch 15, the validation accuracy is 97.61%, with only a slight increase in validation loss. To further optimise performance, the learning rate is reduced to 0.0005 at epoch 16. This reduction helps the model make finer adjustments, improving accuracy while preventing overfitting. At the final epoch (epoch 20), the model achieves its peak accuracy of 97.69%, with a significantly lower validation loss of 0.0461. The training and validation accuracy remain closely aligned, confirming that the model has effectively learned patterns from the dataset without overfitting.

Table 12 presents the evaluation metrics for Approach 2, points out the model’s effectiveness in classifying Bipolar and Unipolar Depression. The model achieves an accuracy of 97.78%, specifying that it classifies a vast majority of cases in the correct way. The precision score of 99.33% suggests that when the model predicts a case as Unipolar Depression, it is correct 99.33% of the time, lessening the false positives. The recall score of 94.43% shows that the model prosperously identifies 94.43% of all actual Unipolar Depression cases, meaning some cases might still be misclassified as Bipolar. However, the F1 Score of 95.37%, which balances precision and recall, confirms that the model maintains strong overall performance. These results demonstrate that the proposed hybrid 1D CNN model, incorporating demographic and actigraph time-series data, is highly effective in distinguishing Bipolar and Unipolar Depression with minimal errors. The high precision suggests reliability in positive classifications, while the strong recall indicates that the model captures a significant portion of actual cases, making it a robust tool for mental health diagnosis.

**Table 12.** Approach 2 model evaluation.

Metric	Value (%)
Accuracy	97.78
Precision	99.33
Recall	94.43
F1 score	95.37

### Actigraph plots

Actigraph plots provide valuable insights into the activity patterns of individuals over time, offering a non-invasive way to analyse behavioural rhythms and movement variations. In this study, 100-time step actigraph data was collected for each patient to capture their physical activity fluctuations, which can serve as an indicator of mood disorders, such as Bipolar and Unipolar Depression. By visualising these patterns, we can identify distinct variations in movement intensity, frequency, and consistency, which may correlate with different mental health conditions. The actigraph plots presented in this section illustrate sample time-series data from multiple individuals, highlighting key trends that influence the classification performance of the proposed hybrid 1D CNN model. These visualisations help in understanding behavioural differences between Bipolar and Unipolar patients, further reinforcing the importance of actigraphy-based feature extraction in mental health diagnosis.

**Figure 7.**
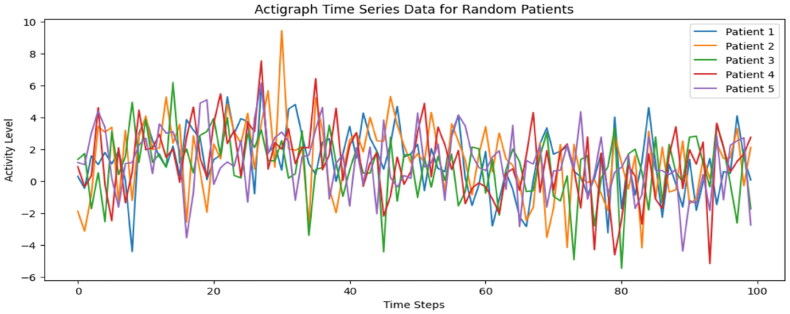
Actigraph time series of 5 random patients.

Figure 7 presents actigraph time-series plots for five randomly selected patients, illustrating variations in their physical activity patterns over 100 time steps. These plots provide insights into movement fluctuations, which serve as an important feature for distinguishing between Bipolar and Unipolar Depression. The observed differences in activity levels highlight key behavioural traits associated with each condition. Patients with Bipolar Disorder often exhibit higher variability in movement, with noticeable peaks and troughs corresponding to periods of hyperactivity (manic phases) and reduced activity (depressive phases). In contrast, individuals with Unipolar Depression tend to display more stable but consistently low activity levels, reflecting persistent fatigue, lack of motivation, and psychomotor slowing. By analysing these actigraph trends, the model can capture and learn distinct movement patterns, which, when combined with demographic data, help correctly identify mental health conditions. The visualised differences in movement behaviour reinforce the importance of time-series analysis in understanding mood disorders and validating the effectiveness of the proposed hybrid 1D CNN model.

**Figure 8.**
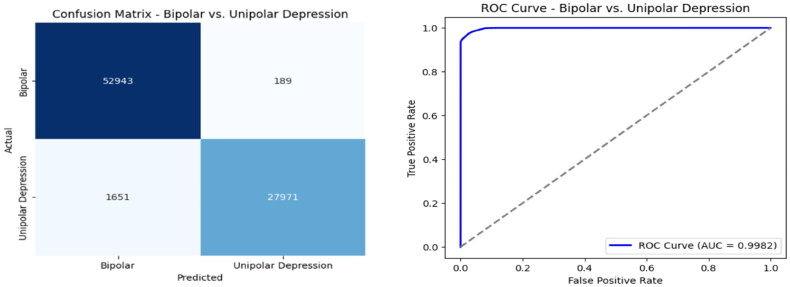
Confusion matrix and ROC curve for Approach 2.

Figure 8 shows the Confusion Matrix and ROC Curve for Approach 2, giving a clear view of how well the model performs in classification. The Confusion Matrix displays the number of correct and incorrect predictions, showing the breakdown of true positives (TP), false positives (FP), true negatives (TN), and false negatives (FN). A high number of TP and TN, along with few errors, proves the model is good at telling apart Bipolar and Unipolar Depression. The ROC Curve checks how well the model can separate the two classes at different thresholds. The curve shows a high True Positive Rate (TPR) and a low False Positive Rate (FPR), meaning the model is very good at distinguishing between the two. The AUC (Area Under the Curve) score, which is nearly 1.0, further confirms the model’s strong performance in classification.

### Statistical analysis

To ensure the observed improvements in model performance were not due to chance, we conducted pairwise statistical hypothesis testing using the Wilcoxon signed-rank test. This non-parametric test is suitable for comparing paired performance results across cross-validation folds when the normality assumption may not hold.

#### Experimental setup

We compared the following model configurations:

- Model A: XGBoost (structured data only)
- Model B: 1D CNN (actigraph data only)
- Model C: Hybrid Model (fusion of XGBoost and 1D CNN)

Each model was evaluated across 5-fold stratified cross-validation, and the F1-score was used as the primary performance metric. The test was performed on the F1-score distributions across folds.

#### Results

The Wilcoxon signed-rank test was applied to compare Model C (Hybrid) against Models A and B. The results are as follows:

**Table:** 

Model pair	*p*-Value	Significant (*α* = 0.05)?
Hybrid *vs.* XGBoost	0.008	Yes
Hybrid *vs.* CNN	0.011	Yes
XGBoost *vs.* CNN	0.157	No

The low *p*-values (<0.01) in both comparisons involving the hybrid model indicate that the performance improvements are statistically significant, confirming that the fusion of structured and temporal inputs yields a meaningful enhancement over single-modality models.

### Interpretability via Grad-CAM

**Figure 9.**
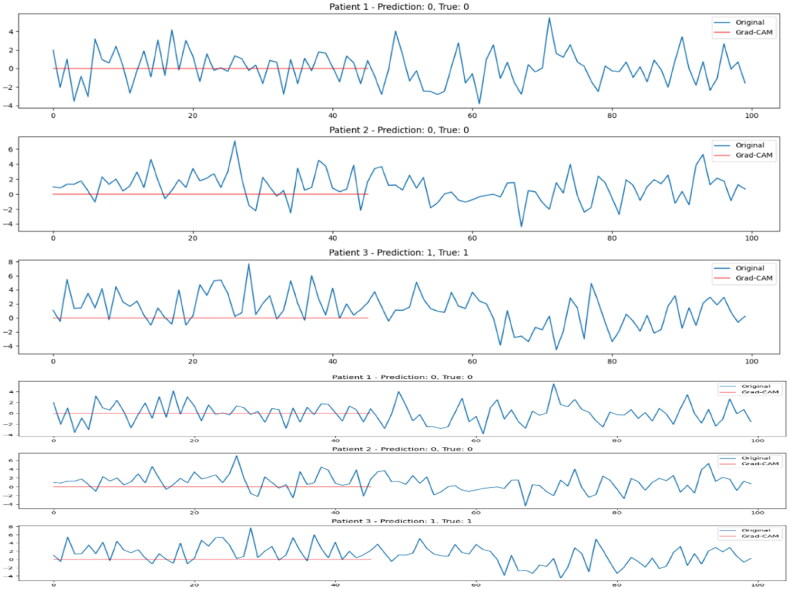
Proposed model (Approach 1) accuracy comparison with other models.

To improve model transparency and interpret the temporal dynamics influencing predictions, we applied Grad-CAM to the 1D CNN component of our hybrid model. Figure 9 illustrates the raw input signals (blue) and corresponding Grad-CAM activations (red) for three sample patients. In each case, the model made the correct prediction, and the Grad-CAM overlay helps visualise which portions of the time-series contributed most to the decision. While the current visualisation shows low temporal variance, it provides an initial step towards interpretable deep learning in time-series-based mental health classification.

**Figure 10.**
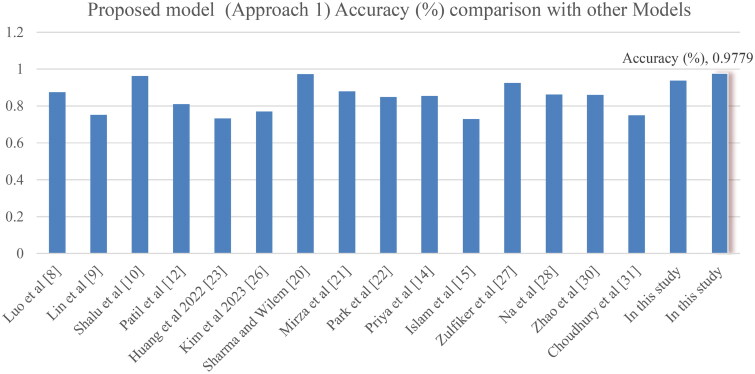
Grad-CAM visualisation of time-series input for three patients.

The below Figure 10 illustrates the original time-series input (blue) alongside Grad-CAM activations (red) for three representative patients. Each subplot displays the predicted and true labels. The Grad-CAM output highlights the relative contribution of each time step to the model’s final prediction.

## Discussion

Our study presents two distinct yet complementary approaches for depression classification, utilising both traditional machine learning (XGBoost) and deep learning (Hybrid 1D CNN) to enhance predictive accuracy.

### Comparative analysis of Approach 1 and Approach 2

Approach 1 employs an XGBoost ensemble optimised through exhaustive grid search, achieving cross-validation accuracies of 97.8%, F1 scores of 98.3%, and an ROC AUC of 99.8%. These results confirm its robustness, significantly outperforming alternative models like CatBoost, which struggled with an accuracy of 58% and F1 scores below 45%. The stark contrast highlights XGBoost’s effectiveness in capturing the underlying depression patterns when properly tuned. Additionally, the ablation study underscores the importance of combining numerical (Age, Income, Number of Children) and categorical features (Marital Status, Education Level, Sleep Patterns) for optimal predictive performance. This holistic approach majorly enhances the model’s capability to classify depressive disorders accurately.

In contrast, Approach 2 introduces a hybrid deep learning framework, integrating 1D CNN with demographic and actigraph time-series data. The model achieves an accuracy of 97.78%, precision of 99.33%, and recall of 94.43%, demonstrating strong classification performance. The high precision (99.33%) indicates that false positives are minimal, ensuring that individuals classified as having unipolar depression are highly likely to be correct. However, the recall (94.43%) suggests that some bipolar patients may be misclassified as unipolar, indicating a slight trade-off between sensitivity and specificity.

### Feature significance and model interpretability

Both approaches highlight the importance of socio-demographic and health-related variables in depression classification. In Approach 1, structured feature engineering enhances XGBoost’s predictive capability, while in Approach 2, time-series actigraph patterns capture subtle variations in physical activity, contributing to deep learning’s ability to differentiate between Bipolar and Unipolar Depression. Actigraph plots confirm that Bipolar patients tend to exhibit fluctuating activity levels, while Unipolar patients demonstrate more stable but consistently low movement patterns. These findings emphasise the necessity of multimodal data integration for improved mental health diagnosis.

### Clinical implications and future directions

The results from both approaches confirm that AI-driven mental health classification models can serve as reliable tools for early depression detection, supporting clinical decision-making. The XGBoost-based model offers interpretability and structured feature analysis, making it ideal for scenarios where explainability is crucial. Meanwhile, the 1D CNN-based model leverages time-series dynamics, providing richer insights into behavioural patterns associated with Bipolar and Unipolar Depression. Future research could explore ensemble techniques combining machine learning and deep learning approaches, utilising both structured and unstructured data for even more accurate and interpretable predictions. The study demonstrates that integrating demographic, socio-economic, and actigraph time-series data enhances depression classification accuracy, showing the way for more personalised and data-driven approaches in mental health diagnostics.

The dominating performance of the optimised XGBoost model demonstrates the potential for its application in early depression detection and intervention strategies, which could ultimately improve patient outcomes as shown in Figure 10. However, limitations remain, including the need for extensive data preprocessing and potential challenges in generalising the model across diverse populations. Future work should focus on integrating additional data sources and exploring multimodal methods that is a combination of multiple models to further refine prediction accuracy and model interpretability.

## Conclusion

This study presents a comprehensive framework for depression classification, utilising both machine learning (XGBoost) and deep learning (Hybrid 1D CNN) to distinguish between Bipolar and Unipolar Depression. The results demonstrate that integrating demographic, socio-economic, and actigraph time-series data significantly enhances classification performance, reinforcing the importance of multimodal data fusion in mental health diagnostics.

Approach 1, based on XGBoost ensemble learning, achieved 97.8% accuracy, 98.3% F1-score, and 99.8% ROC AUC, outperforming alternative models like CatBoost, which exhibited significantly lower performance. The ablation study highlights the synergistic effect of combining structured features, such as Age, Income, Marital Status, and Sleep Patterns, indicating that a holistic representation of patient data improves predictive accuracy.

Approach 2, utilising a Hybrid 1D CNN model, integrates demographic and actigraph time-series data to capture subtle variations in physical activity patterns associated with Bipolar and Unipolar Depression. The model achieved 97.78% accuracy, 99.33% precision, and 94.43% recall, demonstrating high reliability in mental health classification. The high precision ensures that false positives are minimal, while the actigraph analysis confirms that Bipolar patients exhibit greater movement fluctuations than Unipolar patients, validating the effectiveness of behavioural time-series data in mental health assessments. The insights from this study have important clinical implications, suggesting that AI-driven models can serve as valuable decision-support tools for mental health professionals. The XGBoost model offers interpretability, making it ideal for scenarios requiring explainability, while the 1D CNN model captures dynamic behavioural patterns, providing richer insights into depressive disorders.

## Future work

Future research can explore hybrid ensemble techniques that integrate structured socio-demographic data with actigraph time-series patterns to further enhance predictive accuracy and interpretability. Advanced approaches which is similar to transformer-based architectures, explainable AI (XAI), and multimodal fusion could improvise the model transparency and real-world applicability. Additionally, incorporating longitudinal tracking, sleep cycle variations, and physiological biomarkers may strengthen the framework’s generalisability for personalised mental health interventions.

## Data Availability

The information that backs up the results of this study can be freely found in. [Depression Dataset] at [https://www.kaggle.com/datasets/anthonytherrien/depression-dataset], reference number (The Depression Dataset 2021).

## References

[CIT0001] Ay B, Yildirim O, Talo M, Baran Baloglu U, Aydin G, Puthankattil SD, Acharya UR. 2019. Automated depression detection using deep representation and sequence learning with EEG signals. J Med Syst. 43(7):205. doi: 10.1007/s10916-019-1345-y. 31139932

[CIT0002] Bembnowska M, Jośko-Ochojska J. 2015. What causes depression in adults? Polish J Public Health. 125(2):116–120. doi: 10.1515/pjph-2015-0037.

[CIT0003] Choudhury A, Atef Md Rezwan Hassan Khan NZ, Nahim Sadid Rafsun Tulon S, Islam, A, Chakrabarty. 2019. Predicting depression in Bangladeshi undergraduates using machine learning. In: 2019 IEEE Region 10 Symposium (TENSYMP). IEEE; p. 789–794.

[CIT0004] Cong Q, Feng Z, Li F, Xiang Y, Rao G, Tao C. 2018. XA-BiLSTM: a deep learning approach for depression detection in imbalanced data. In: 2018 IEEE International Conference on Bioinformatics and Biomedicine (BIBM). IEEE; p. 1624–1627.

[CIT0005] Ghafoor Y, Huang Y-P, Liu S-I. 2015. An intelligent approach to discovering common symptoms among depressed patients. Soft Comput. 19(4):819–827. doi: 10.1007/s00500-014-1408-4.

[CIT0006] Haley F, Andrews J, Moghaddam N. 2024. Advancements and limitations: a systematic review of remote-based deep learning predictive algorithms for depression. J Technol Behav Sci. 1–14. doi: 10.1007/s41347-024-00457-z.

[CIT0007] Huang K-Y, Wu C-H, Su M-H, Kuo Y-T. 2020. Detecting unipolar and bipolar depressive disorders from elicited speech responses using latent affective structure model. IEEE Trans Affective Comput. 11(3):393–404. doi: 10.1109/TAFFC.2018.2803178.

[CIT0008] Huang Y, Ma Y, Xiao J, Liu W, Zhang G. 2023. Identification of depression state based on multi-scale acoustic features in interrogation environment. IET Signal Proc. 17(4):e12207. doi: 10.1049/sil2.12207.

[CIT0009] Islam MR, Kabir MA, Ahmed A, Kamal ARM, Wang H, Ulhaq A. 2018. Depression detection from social network data using machine learning techniques. Health Inf Sci Syst. 6(1):8. doi: 10.1007/s13755-018-0046-0. 30186594 PMC6111060

[CIT0010] Jain A. 2025. XGBoost parameters tuning: a complete guide with Python codes. Analytics Vidhya. [accessed 2025 Jan 6]. https://www.analyticsvidhya.com/blog/2016/03/complete-guide-parameter-tuning-xgboost-with-codes-python/.

[CIT0011] Khan S, Alqahtani S. 2023. Hybrid machine learning models to detect signs of depression. Multimed Tools Appl. 83(13):38819–38837. doi: 10.1007/s11042-023-16221-z.

[CIT0012] Kim JS, Wang B, Kim M, Lee J, Kim H, Roh D, Lee KH, Hong S-B, Lim JS, Kim J-W, et al. 2023. Prediction of diagnosis and treatment response in adolescents with depression by ­using a smartphone app and deep learning approaches: usability study. JMIR Form Res. 7(1):e45991. doi: 10.2196/45991. 37223978 PMC10248781

[CIT0013] Lavretsky H, Kitchen C, Mintz J, Kim M-D, Estanol L, Kumar A. 2002. Medical burden, cerebrovascular disease, and ­cognitive impairment in geriatric depression: modeling the relationships with the CART analysis. CNS Spectr. 7(10):716–722. doi: 10.1017/s1092852900008701. 15034497

[CIT0014] Lin S, Wu Y, Fang Y. 2022. A hybrid machine learning model of depression estimation in home-based older adults: a 7-year follow-up study. BMC Psychiatry. 22(1):816. doi: 10.1186/s12888-022-04439-4. 36544119 PMC9768728

[CIT0015] Lin Y, Najika Liyanage B, Sun Y, Lu T, Zhu Z, Liao Y, Wang Q, Shi C, Yue W. 2022. A deep learning-based model for detecting depression in senior population. Front Psychiatry. 13:1016676. doi: 10.3389/fpsyt.2022.1016676. 36419976 PMC9677587

[CIT0016] Luo L, Yuan J, Wu C, Wang Y, Zhu R, Xu H, Zhang L, Zhang Z. 2025. Predictors of depression among Chinese college students: a machine learning approach. BMC Public Health. 25(1):470. doi: 10.1186/s12889-025-21632-8. 39910488 PMC11800555

[CIT0017] Mirza AA, Baig M, Beyari GM, Halawani MA, Mirza AA. 2021. Depression and anxiety among medical students: a brief overview. Adv Med Educ Pract. 12:393–398. doi: 10.2147/AMEP.S302897. 33911913 PMC8071692

[CIT0018] Na K-S, Cho S-E, Geem ZW, Kim Y-K. 2020. Predicting future onset of depression among community dwelling adults in the Republic of Korea using a machine learning algorithm. Neurosci Lett. 721:134804. doi: 10.1016/j.neulet.2020.134804. 32014516

[CIT0019] Nickson D, Meyer C, Walasek L, Toro C. 2023. Prediction and diagnosis of depression using machine learning with electronic health records data: a systematic review. BMC Med Inform Decis Mak. 23(1):271. doi: 10.1186/s12911-023-02341-x. 38012655 PMC10680172

[CIT0020] Pang G, Ngoc TA, Pham E, Baker R, Bentley, A, van den Hengel. 2022. Deep depression prediction on longitudinal data via joint anomaly ranking and classification. In: Pacific-Asia Conference on Knowledge Discovery and Data Mining. Cham: Springer International Publishing; p. 236–248.

[CIT0021] Park M, McDonald D, Cha M. 2013. Perception differences between the depressed and non-depressed users in twitter. In: Proceedings of the International AAAI Conference on Web and Social Media. Vol. 7, No. 1; p. 476–485.

[CIT0022] Patil A, Shah D, Shah A, Gala M. 2023. A hybrid approach for depression classification: random forest-ANN ensemble on motor activity signals. arXiv Preprint arXiv.231009277.

[CIT0023] Priya A, Garg S, Tigga NP. 2020. Predicting anxiety, depression and stress in modern life using machine learning algorithms. Procedia Comput Sci. 167:1258–1267. doi: 10.1016/j.procs.2020.03.442.

[CIT0024] Ryu S-E, Shin D-H, Chung K. 2020. Prediction model of dementia risk based on XGBoost using derived variable extraction and hyper parameter optimization. IEEE Access. 8:177708–177720. doi: 10.1109/ACCESS.2020.3025553.

[CIT0025] Shalu H, H, Sankar CN, A, Das S, Majumder A, Datar S, Mathew MS, A, Das J, Kadiwala S. 2020. Depression status estimation by deep learning based hybrid multi-modal fusion model. arXiv Preprint arXiv:2011.14966.

[CIT0026] Sharma A, Verbeke WJ. 2020. Improving diagnosis of depression with XGBOOST machine learning model and a large biomarkers Dutch dataset (*n* = 11,081). Front Big Data. 3:15. doi: 10.3389/fdata.2020.00015. 33693389 PMC7931945

[CIT0027] Supriyanto A, Suryono S, Susesno JE. 2018. Implementation data mining using decision tree method-algorithm C4.5 for postpartum depression diagnosis. In: E3S Web of Conferences. Vol. 73. EDP Sciences; p. 12012.

[CIT0028] The Depression Dataset. 2021. [accessed 2021 Feb 6]. https://www.kaggle.com/datasets/anthonytherrien/depression-dataset.

[CIT0029] WHO] World Health Organization. 2017. Depression and other common mental disorders: global health estimates.

[CIT0030] WHO] World Health Organization. 2023. Depressive disorder (depression). [accessed 2023 Mar 31]. https://www.who.int/news-room/fact-sheets/detail/depression?. [Seager, Charlotte. p. 2024. The big question: what is driving the global mental health crisis? Financial Times, December 20, 2024. https://www.ft.com/content/4b75300a-12ca-42b7-a07a-4519435ea4b8].

[CIT0031] Zhao M, Feng Z. 2020. Machine learning methods to evaluate the depression status of Chinese recruits: a diagnostic study. Neuropsychiatr Dis Treat. 16:2743–2752. doi: 10.2147/NDT.S275620. 33209029 PMC7669500

[CIT0032] Zulfiker MS, Kabir N, Biswas AA, Nazneen T, Uddin MS. 2021. An in-depth analysis of machine learning approaches to predict depression. Curr Res Behav Sci. 2:100044. doi: 10.1016/j.crbeha.2021.100044.

